# Non-invasive prenatal testing for ‘non-medical’ traits: ensuring consistency in ethical decision-making

**DOI:** 10.1080/15265161.2021.1996659

**Published:** 2021-11-30

**Authors:** Hilary Bowman-Smart, Christopher Gyngell, Cara Mand, David J. Amor, Martin B. Delatycki, Julian Savulescu

**Affiliations:** 1 Department of Paediatrics, University of Melbourne, Parkville, Australia; 2 Murdoch Children’s Research Institute, Parkville, Australia; 3 Victorian Clinical Genetics Services, Parkville, Australia; 4 Uehiro Centre for Practical Ethics, University of Oxford, Oxford, United Kingdom

## Abstract

The scope of non-invasive prenatal testing (NIPT) could expand in the future to include detailed analysis of the fetal genome. This will allow for the testing for virtually any trait with a genetic contribution, including ‘non-medical’ traits. Here we discuss the potential use of NIPT for these traits. We outline a scenario which highlights possible inconsistencies with ethical decision-making. We then discuss the case against permitting these uses. The objections include practical problems; increasing inequities; increasing the burden of choice; negative impacts on the child, family, and society; and issues with implementation. We then outline the case for permitting the use of NIPT for these traits. These include arguments for reproductive liberty and autonomy; questioning the labelling of traits as ‘non-medical’; and the principle of procreative beneficence. This summary of the case for and against can serve as a basis for the development of a consistent and coherent ethical framework.

## Introduction

It is the year 2030, and an expectant parent visits her clinician to request non-invasive prenatal testing (NIPT). At this time, the scope of NIPT has expanded: the test can now reveal much more information than is currently possible. She meets with the clinician, who says the test can tell her if the fetus has a high chance of having trisomy 21 or other common aneuploidies (a different number of chromosomes than typical). The clinician then asks her what else she would like to know. He explains that the test can also give information about single-gene disorders and variants such as pathogenic *BRCA1* variants. Furthermore, he then says that they can examine the genome of the fetus and provide information about general risk for diseases such as diabetes and schizophrenia. Beyond this, the same approach can be used to screen for information about things such as reduced cognitive ability, even if this will not necessarily cause intellectual disability. The mother takes some time to consider the many choices before her.

Although this scenario is hypothetical, there are currently companies aiming to offer pre-pregnancy testing services for genetic risk for polygenic traits (e.g. diabetes, some cancers), such as Orchid pre-conception screening (Branca 2021) or Genomic Prediction’s embryo screening (Regalado 2019). Here we discuss an extension of these practices to the choice this mother has been given – using genetic sequencing and approaches like polygenic scores to screen for ‘sub-clinical’ or ‘non-medical’ traits.

NIPT uses a sample of the gestational parent’s blood to analyse cell-free fetal DNA (cffDNA). Currently, it can screen for aneuploidies such as trisomy 21, as well as sex chromosome aneuploidies (SCAs) (Gil et al. 2017), and in some tests, microdeletions (Kucharik et al. 2020). It can also be used to determine the fetal sex (Bowman-Smart et al. 2020). NIPT can be done early in gestation (at the end of the first trimester) and is more accurate for diagnosing aneuploidies than other prenatal screening methods used in that gestational period, such as first trimester combined screening (McLennan et al. 2016). However, NIPT is a screening test, and is not diagnostic. The accuracy of NIPT varies for different conditions; for example, it is more accurate for trisomy 21 than SCAs (Deng, Cheung & Liu 2021). Although rare, false positives and false negatives can occur; one multicentre study found a false positive rate of 0.095% and a false negative rate of 0.006% (Suzumori et al. 2021). Furthermore, test failure can happen (Yaron 2016). It is recommended that any high-risk result be followed up with invasive testing such as chorionic villus sampling (CVS) or amniocentesis to confirm the diagnosis (Skrzypek and Hui 2017). NIPT is likely to become more widely available for single-gene disorders such as achondroplasia (Skrzypek and Hui 2017). Furthermore, there is the potential for developments in cell-based methods of NIPT to provide more accurate information (Vestergaard et al. 2017, Jeppesen et al. 2021). In the future, the range of conditions that can be screened for by NIPT is expected to expand towards providing detailed analysis of the fetal genome (Srebniak et al. 2020).

We are moving into a new era of increased prenatal screening and diagnosis. Prospective parents could soon access more information about their future child then they have before. Some of this information is already available through diagnostic techniques such as amniocentesis or CVS. However, NIPT significantly lowers the barriers for accessing this information, can be implemented as a population screening program, and may be ‘routinized’ in a way that impacts informed choice (Cernat et al. 2019). Most importantly, NIPT may also lower the barrier to termination of pregnancy (TOP) by providing information earlier in the pregnancy; TOP can become more challenging at later gestations (Janiak et al. 2014).. Whole genome analysis offers the prospect of vast amounts of predictive information (not currently provided in other prenatal screening tests), including about non-medical traits. This will affect use of CVS and amniocentesis, but its effect will be most pronounced in NIPT. Therefore, in this article, we focus on the context of NIPT, though our arguments largely extend to amniocentesis and CVS. We must think carefully about what kind of information should be available, and how we should present it. In doing so, we must evaluate exactly how we are reaching these decisions, and what criteria we are using. Previous ethical frameworks and guidelines are not sufficient for the sheer volume of information that we will be able to generate and may result in inconsistent approaches.

Here, we explore the ethical issues raised by – and the positions for and against – the use of emerging prenatal screening technologies for traits which cross the disease-health boundary, such as cognitive ability. For brevity, we will call these “non-medical traits”, although we also discuss the framing of such traits in terms of pathology, as frequently they will constitute ‘disease’ or ‘disorder’ if outside of the normal distribution (this applies to cognitive ability, height, vision, hearing, and so on.). Although this article is structured as a ‘case for’ and ‘case against’, the ethical arguments are critically discussed (including possible responses where relevant).

There are complex moral questions around what it should be permissible to screen for, whether such information should be offered or made available upon request, what uses should be publicly funded, and whether there should be restrictions placed on the commercial direct-to-consumer (DTC) sector. The debate about what should be included in prenatal screening should not be bounded by simply accepting what is or is not possible.

Information about non-medical traits might be provided through testing for specific variants or chromosomal changes. For example, the 15q11.2 microdeletion involving *NIPA1* decreases IQ by an average of 4.3 points, which would be considered a sub-clinical impact on cognitive ability, although it also slightly increases the chance of intellectual disability (Jønch, Douard et al. 2019). It may also be possible to use polygenic scores – a score calculated from the contribution of many genetic variants – to predict a variety of non-medical traits such as cognitive ability, creativity, height and appearance (Lewis and Green 2021, Munday and Savulescu 2021). Polygenic scores for medical conditions such as diabetes and heart disease are frequently discussed in the clinical context as part of a move towards ‘personalized medicine’ (Yanes et al. 2020); here, we focus on their use for non-medical traits.

It is important to note that the utilization of polygenic scores to predict a variety of traits is currently of limited use for a variety of reasons that will be explored (Duncan et al. 2019, Martin et al. 2019). Although proponents of the use of polygenic scores emphasise their potential, it may be the case that even with further research, they are ultimately not useful in reproductive decision-making. Nonetheless, it is important to assess the ethical issues associated with emerging technologies before they become possible, even if some of these technologies do not mature. It is also important to note that these technologies may come into use even if they remain relatively unreliable, particularly in the DTC sector, if there is consumer demand for such a test and companies oversell test performance.

In this paper, we examine some of the current inconsistencies facing the landscape of prenatal genetic screening, and why the use of technologies such as NIPT to screen for non-medical traits is not completely at odds with our current approaches. To resolve these inconsistencies, we need a clearer ethical framework. Here, we do not aim to propose such a framework, but instead establish the lay of the land of these ethical issues. We will examine the arguments against using NIPT for non-medical traits, including lack of certainty, possible inequities, the impact on informed choice, and the impact on the parent-child relationship. We will then examine the arguments in favour of using NIPT for non-medical traits, which include an emphasis on autonomy in reproductive decision-making, a questioning of the disease-health distinction, and the principle of procreative beneficence.

## Current standards and inconsistencies

Decisions about screening programs are generally guided by the Wilson and Jungner criteria, devised in 1968 (Wilson and Jungner 1968). First amongst these is that the condition should be an important health problem. Other criteria include that the test should be suitable and accurate, and that the condition should be treatable. Although these criteria are not recent, they persist broadly into modern screening criteria (Dobrow et al. 2018).

However, prenatal screening programs have different goals to other kinds of population screening programs (de Jong et al. 2011). The goal of population screening programs is frequently to reduce the incidence, morbidity or mortality of a disease within a given population (World Health Organization 2020). However, the stated goal of prenatal screening for trisomy 21, for example, is not to ‘prevent’ the genetic condition or treat it in existing individuals of a population. Instead, by facilitating ‘informed choice’ (World Health Organization 2020) it determines which individuals come into existence. Although *in utero* therapies may become an option in the future, the current goal of prenatal screening is generally to ‘improve outcomes’ for women and families (Dukhovny & Norton 2018). Alberry et al. (2021, 427) state that “NIPT’s ultimate aim should be facilitating prenatal decisions surrounding autosomal aneuploidies”. Prenatal screening can allow parents to use information to make decisions, such as making informed reproductive choices (although in Germany, the justification for public funding of NIPT is specifically restricted solely to reducing rates of invasive testing (Rehmann-Sutter & Schües 2020)). The aim of prenatal screening is subject to some debate; de Jong and de Wert (2015) discuss the view that population prenatal screening programs are really about reducing the incidence of disability, and argue that such programs are only morally justified if the aim is to provide options for meaningful reproductive choices.

Thus, if parents choose, for example, to terminate the pregnancy, they are preventing the existence of a person with a genetic condition rather than preventing the genetic condition itself. Prenatal screening, more so than other programs such as newborn screening, is about ‘personal utility’ as much as, if not more so, than clinical utility. Personal utility is a complex concept. In a 2017 systematic review, Kohler et al. (2017) identified four key domains (three relating to personal outcomes, and one relating to social outcomes). The domains relating to personal outcomes included affective (impact on the person’s emotional state), cognitive (the value of things such as self-knowledge) and behavioural (impact on, for example, the person’s decision-making). The social domain includes outcomes that impact a person’s interpersonal relationships or other factors such as privacy concerns. One of the key highlighted areas of the behavioural domain is reproductive decision-making, which is a utility directly related to the decision-maker rather than the fetus.

However, prenatal screening can be also viewed as having clinical utility because it can inform decision-making around a particular medical procedure (TOP), which may be considered treatment in a sense. Information derived from NIPT is frequently used for the purposes of selective TOP, although the rates of selective TOP may not differ much from other prenatal screening methods (Hill et al. 2017). There are significant and complex debates around the ethics of TOP itself; however, these debates (such as the moral status of the fetus) are outside the scope of this paper.

The use of prenatal screening for personal utility rather than clinical utility is reflected in the ways in which prenatal screening differs from other kinds of screening programs. For example, the criterion that the condition should be treatable is not applicable to prenatal screening. Indeed, in the prenatal setting where selective TOP is an option, there may be even more reason to screen for non-treatable conditions. This leads us to question of how an approach designed to fit other screening programs can be adjusted for prenatal screening, and what other criteria we must call into question.

When discussing the ethical considerations around the use of NIPT for non-medical traits, it is also necessary to consider the different contexts in which this testing might be offered. It is important to note that prenatal screening technologies such as NIPT may be implemented (and currently are, in many parts of the world) as user-pays testing, rather than as part of a government-funded population screening program (Gadsbøll et al. 2020). Where NIPT is implemented as part of a publicly funded population screening program, the information provided and criteria for screening vary between countries. For example, some countries who have implemented public funding of NIPT restrict the information provided to a small number of conditions: France only funds NIPT for trisomy 21, and funding through the National Health Service in the UK only covers trisomies 13, 18 and 21. Information about sex, sex chromosome aneuploidies, and other chromosomal anomalies is not provided (Public Health England 2021, Gadsbøll et al. 2020). Questions around public funding form part of the ethical questions that will be discussed. The use of NIPT as population screening includes additional considerations, such as resource-allocation. Therefore, it is important to distinguish between ethical issues related to provision of such information through NIPT in general, and provision of such information through NIPT in a population screening program.

However, although we have just focused on trisomies 13, 18 and 21 as examples, we must also consider other traits screened for by NIPT. Other current uses of NIPT also highlight inconsistencies with traditional screening criteria. For example, although it is not its primary purpose, NIPT is already widely available to test for sex, which is a non-medical trait, even if this is framed as an ‘incidental finding’ from screening the sex chromosomes (Bowman-Smart et al. 2020). Concerns have been raised about the use of NIPT for sex-determination, such as the possibility of facilitating sex-selective TOP (Chapman and Benn 2013). However, evidence suggests that there is broad support amongst consumers for providing NIPT for sex-determination (Bowman-Smart et al. 2019a). One may argue that sex determination is in effect a ‘incidental finding’, but our previous survey of Australian women found that 31% of respondents indicated that determining fetal sex was a reason they pursued NIPT (Bowman-Smart et al. 2019b). These reasons usually relate to perceived personal utility, such as a perception that this knowledge is useful for planning and preparation, enhancing maternal-fetal bonding, and bestowing a sense of personhood upon the fetus (Shipp et al. 2004, Barnes 2013) If we continue to provide sex-determination through NIPT because consumers perceive it as having personal utility, we should recognise that this is testing for a non-medical trait.

NIPT currently also screens for conditions that may not be considered ‘serious’ health problems. There is an SCA that can be identified by NIPT called triple X syndrome (47,XXX). In the case of 47,XXX, the children are females and have an extra X chromosome. This aneuploidy results in an average reduction of 20 intelligence quotient (IQ) points (Otter, Schrander-Stumpel and Curfs 2010); this does not usually result in an intellectual disability. Otherwise, the symptom profile is mild enough that only a small minority of individuals with 47,XXX ever come to clinical attention (Wigby et al. 2016). The primary physical trait is increased height, and there is also an increased risk for psychiatric disorders such as depression (Wigby et al. 2016). A 2019 Danish nationwide cohort study found that 87% of females with 47,XXX are undiagnosed and the incidence is likely to be higher than previously thought (Berglund et al. 2019).

Decision-making following a prenatal diagnosis of 47,XXX varies in different regions of the world. In a 2018 study in the United States, many women (66%) did not proceed to diagnostic testing following an increased chance result for SCA and genetic counselling (Ramdaney, Hoskovec et al. 2018); however, in a 2019 study from China, the majority of women did proceed to diagnostic testing and the TOP rate for 47,XXX was 27%. This is lower than other SCAs such as Turner syndrome (45,X) (Xu et al. 2019). Similarly, a 2012 review of decision-making following prenatal diagnosis of an SCA found that 47,XXX was the SCA most associated with continuation of pregnancy (68%) despite having the highest impact on IQ reduction of the four most common SCAs, 45,X, 47,XXX, 47,XXY and 47,XYY – this implies that possibly up to one third elect to terminate the pregnancy (Jeon, Chen and Goodson 2012, Printzlau, Wolstonecraft and Skuse 2017).

There are two further important features of 47,XXX that make it relevant to our discussion. First, it has a mild enough symptom profile that the impact is often sub-clinical, although of course sometimes it will be clinical. Second, some parents nonetheless use this information to make a particular reproductive choice (selective TOP). What is it about 47,XXX that leads parents to make these decisions? One trait that may provide a reason is the likely negative impact on cognitive ability. If this is the case, parents may be proceeding from one of two reasons: first, that the average impact of 47,XXX on cognitive ability, even if it does not result in intellectual disability, is undesired; or second, that the parents have a low tolerance for increasing the risk of intellectual disability. It may also be a combination of these two reasons. It is also possible that the risk of psychiatric disorder or increased height motivates some choices, but it is unlikely that they are the main reasons in all choices.

It is possible that the reason 47,XXX is reported by some NIPT providers is because it is ‘incidental’ information to screening for other, potentially perceived as more serious, SCAs such as Turner syndrome. Thus, the question may be whether 47,XXX is an ‘incidental finding’ that warrants being returned to the prospective parents. However, if the answer to that question is yes, then there seems no reason why it should not also be morally permissible to screen for it directly, excluding questions of resource allocation. The question of whether it is relevant information for the parents to use for decision-making remains the same regardless of the source of the information.

It is not a sufficient reason to screen for 47,XXX simply because it is an aneuploidy and easy to screen for. As we have stated, decision-making around what to offer for prenatal screening is not just a technical question, it is an ethical one. Controversies within professional societies about whether to screen for SCAs such as 47,XXX have thus far focused primarily on technical questions of test performance (Christiaens, Chitty & Langlois 2021). These facts are relevant to the ethical discussion, but they are only one piece of the puzzle. A consistent approach to decision-making around prenatal screening should focus on the nature of the phenotype, rather than the genotype. The phenotype of 47,XXX, in most cases, does not result in serious clinical problems. We must then examine what would be the implications of screening for aspects of the phenotype of 47,XXX (e.g. sub-clinical decrease in cognitive ability), even when it is not attributable to a variant or chromosomal change classified as pathological.

This is because it is not the genotype of an individual that matters, it is the phenotype. It is persons and their minds which matter, not some fact about biology, including genes and chromosomes. Our biology is only instrumentally valuable to our identity, autonomy and well-being. The exact genetic basis of a condition is not morally relevant. What is morally relevant is the impact that genetic changes have on an individual in their everyday life. If there were some large chromosomal change that did not impact on an individual as they go about their life, how they view themselves, or their level of well-being, there would be no reason to screen for it.

Therefore, if it is acceptable to screen for 47,XXX on the basis that the phenotype has a sufficiently negative impact on the life of the child – if it is the phenotype that is morally relevant – then presumably it is acceptable to screen for traits associated with the phenotype, such as a decrease in cognitive ability, increased risk of depression, and increased height. If parents seek out 47,XXX screening on the basis that they are concerned about the sub-clinical impact on cognitive ability, and this is acceptable, then it should also be acceptable for them to seek out screening for similar impacts on cognitive ability that have other genetic bases. Conversely, if it is not acceptable to screen for these traits themselves, then it should not be acceptable to screen for 47,XXX.

The basis of a sub-clinical impact on a trait such as cognitive ability – beyond issues of accuracy – has no relevance. It is the trait that matters. We therefore must either accept screening for these traits, or not accept screening for genetic variants classified as pathological that result in those same traits. One must fall one on side of the fence. We now present the cases for and against accepting screening for non-medical traits using NIPT.

### The case against

We will now examine some of the arguments against using NIPT for non-medical traits, such a sub-clinical impact on cognitive ability. We will not cover all of them, as there is a significant amount of relevant literature. Instead, we aim to identify and critically discuss some key ethical arguments that may relate specifically to the use of NIPT for non-medical traits.

#### Polygenic scores and practical problems

First, there is an objection grounded in practicality, which argues that genetics will only ever be a poor predictor of non-medical traits such as cognitive ability, and that NIPT will never be accurate enough to be useful for decision-making. The validity of polygenic scores as they are currently calculated and used has been criticised (Janssens 2019). Polygenic scores tend to be computed using data from overwhelmingly European populations, and thus are less predictive in other human populations (Duncan et al. 2019). Thus, the inappropriate use of polygenic scores in the clinical setting has the potential to exacerbate disparities and inequalities (Martin et al. 2019).

Furthermore, it is important to remember that polygenic scores demonstrate an association, rather than a causal relationship between the gene variants and the trait (Crouch and Bodmer 2020); without appropriately establishing a causal relationship, one would be facing the prospect of terminating a pregnancy based on a probability due to association alone. Knowing that there is an association may be enough for some parents, but not for others. For example, it may be relevant to reproductive decision-making if, theoretically, parents knew an association between a polygenic score and decreased cognitive ability was mediated by a third variable, which was, perhaps, a propensity to head injuries, or a metabolic deficiency, causing the decreased cognitive ability; the parents may thus be more interested in undertaking measures to prevent the head injuries or metabolic deficiency, rather than terminating the pregnancy. This course of action would also result in the desired outcome (avoidance of a child with decreased cognitive ability). That third variable might also be entirely environmental. For example, if there were widespread discrimination against a group of people with a particular trait, and this discrimination resulted in them being exposed to higher amounts of lead which in turn impacted their cognitive ability, the genetic basis for that particular trait would be associated with decreased cognitive ability even though the actual direct cause is lead exposure, itself caused by discrimination. Furthermore, the association may simply be entirely spurious. Thus, understanding causality is important. Establishing causality would also be useful for dealing with issues such as confounding or selection bias (Huang and Labrecque 2019).

Another practical problem with the use of polygenic scores for screening purposes is that through the process of pleiotropy – where one gene or set of genes affects multiple traits – a polygenic score for one trait may also predict another. This may, for example, mean that a polygenic score for schizophrenia is also associated with other related traits such as anxiety (Zheutlin et al. 2019), or a polygenic score for epilepsy being associated with neuroticism-related personality traits (Leu et al. 2020). When it comes to non-medical traits, we might also consider that polygenic scores for schizophrenia and bipolar disorder are also associated with creativity (Power et al. 2015, Greenwood 2020). Thus selecting against schizophrenia may decrease the probability of creativity. Of course, these statements are not necessarily true and are dependent on, as previously stated, the establishment of a causal relationship between the gene variants and the traits in question, and the *direction* of that causal relationship. However, the key issue is that when screening a fetus for a polygenic score for height or cognitive ability, parents may also be inadvertently selecting against other traits, which may in fact be desirable to them and beneficial to the future child.

Furthermore, Ravitsky et al. (2017) argue that in general, any approach to NIPT that utilises whole genome sequencing is likely to result in a higher number of false positives and false negatives compared to actionable results. Although, as previously discussed, the technology is developing, NIPT is still regarded as a screening test, and results must be confirmed by invasive diagnostic testing. If a whole-genome approach is taken, the information that is generated is of such breadth that any pregnancy is likely to be given information that prospective parents may want to confirm through invasive testing, although they may choose not to do so. The difficulty of ‘information overload’ is well recognised in the literature around informed consent in genomics (Bunnik et al. 2021). All fetuses are likely to have an increased chance of some disposition to disease, limitation or other trait. Thus, one of the key attractive points about NIPT – its non-invasiveness – may no longer hold, if nearly every test provides results that should be confirmed by invasive testing. If the results from whole-genome NIPT cannot provide a reliable basis for decision-making, then they may simply not be useful. Furthermore, this may create a burden on resources, particularly if this approach is used in the public system.

Therefore, a key criticism of the use of NIPT for non-medical traits is that the technology simply may not develop to the point where it can provide meaningful or useful information for reproductive decision-making. Unlike the literature discussing the use of polygenic scores in pre-implantation genetic testing (Treff et al. 2020, Munday and Savulescu 2021), where the technology may be used to select between several different embryos, and some embryos will not be chosen regardless, the use of NIPT and polygenic scores means that the parents will be making a decision between having or not having one *particular* fetus. Mauron (2015, 570) thus describes NIPT as “a kind of self-limiting technology” compared to embryo selection. TOP is likely to be more psychologically impactful than choosing between different embryos, for example partly because TOP is a stigmatised procedure, and the perceived stigma is predictive of increased psychological distress (Steinberg et al. 2016, Hanschmidt et al. 2016). Work by Lou et al. (2018) found that couples described the process of TOP following a prenatal diagnosis as ‘existentially burdensome’, due to “fear of regret and concern about ending a potential life”. Thus, parents may require a stronger evidence base for such a decision. If polygenic scores cannot reach high enough predictive values to provide significant personal or clinical utility for prenatal screening, then this would be an argument against their use.

However, while we can recognise the current limitations of the use of polygenic scores and NIPT in general, we cannot ignore the possibility of their implementation in the future. It is difficult to say if or when polygenic scores for non-medical traits may become useful enough for prenatal screening. On the optimistic side, Harden (2021) argues that they are beginning to ‘rival traditional social science variables’. However, Turkheimer (2015) argues that due to the complexity of the variables at play, some of the key problems associated with polygenic scores are likely to never be solved; and indeed, even though he made this statement six years ago, his points remain relevant and convincing.

However, as we have pointed out in the introduction, commercial companies are already offering selective reproductive technologies based on polygenic scores. Given that NIPT is already a test with multiple ‘brands’ that are often discussed in a way similar to consumer products (Crabbe, Stone & Filoche 2019), it is not difficult to foresee the introduction of these approaches into the space of prenatal screening. To ignore the use of reproductive technologies for non-medical traits simply because it is not ‘possible yet’ opens us up to an ethical void if or when it does become possible – or is made available regardless of accuracy, by commercial institutions with fewer ethical scruples. Thus, we must address further arguments against the use of NIPT for non-medical traits.

#### Increasing inequities

Another concern relating to permitting the use of NIPT to test for non-medical traits is that it may result in increased inequities between those who can access the test and those who cannot (or are not willing to use it). Currently, although NIPT is publicly funded in some countries such as Belgium, it is only available on a user-pays basis in other countries such as the United States and Australia (Van Elslande et al. 2019, Gadsbøll et al. 2020). Women who access NIPT in these user-pays contexts are more likely to be highly educated, of higher socio-economic status, and older (Hui et al. 2018). Thus, a concern is that expanding the scope of NIPT, without concurrent expansion of access, means that parents with means will be able to select their children and those without the means will not. This may result in a divide between the genetic ‘haves’ and ‘have-nots’. Furthermore, on a global level, concerns have previously been raised about the relationship between genetics and genomics and existing global health inequalities (Gibbon, Kilshaw & Sleeboom-Faulkner 2018). This concern about inequality from reproductive technologies, genetic selection and other related technologies such as gene editing have been raised previously in surveys and ethical literature (Gammeltoft and Wahlberg 2014, van Dijke et al. 2018, van Dijke et al. 2021).

We might also consider the fact that if NIPT becomes publicly funded for SCAs such as 47,XXX, but not for things that are considered ‘non-medical’ such as polygenic scores for cognitive ability – our previous survey indicated a lack of support for public funding for NIPT for non-medical traits (Bowman-Smart et al. 2019b) – then this in turn may result in inequality. If women who have the means have the option of screening for a broader range of causes of sub-clinical impact on cognitive ability, and women who do not have the means only have the option of screening for few causes, this may in turn result in inequality.

Nonetheless, the various ethical concerns discussed in this paper, as well as questions of resource allocation, may mean that information from NIPT about ‘non-medical’ traits is not covered by a publicly funded population screening program. Indeed, as has been noted, the National Health Service in the UK does not provide information on traits such as sex (Public Health England 2021). Belgium explicitly decided not to include SCAs as part of their public funding of NIPT, although this was because of concerns about validity and clinical utility, rather than ethical concerns (Van Den Bogaert et al. 2021). Therefore, while there are concerns about restriction of information from publicly funded tests and inequality, it is important to recognise that (rightly or wrongly) there is precedent in population screening programs for not providing information that is accessible through user-pays NIPT.

#### Increasing the burden of choice

Expanding the scope of NIPT to include a wider range of traits, including non-medical traits, means that parents are likely to be faced with an increased number of choices to make. Once concern is that increasing the number of choices parents must make could result in ‘decision fatigue’, where repeated acts of decision-making result in an impaired ability to make further decisions (Pignatiello, Martin and Hickman Jr 2020). Furthermore, there have also been critiques of how valuable choice in fact is in the context of healthcare (Zolkefli 2017). Therefore, the personal utility of NIPT may not be realised.

Pregnant women often make decisions in conjunction with their partner, and some include other family members in the decision-making process (Wätterbjörk et al. 2015). They may also involve healthcare professionals, asking them for their perspective on what decision to make (Hertig et al. 2014). Pregnant women develop different methods of coping with probabilities generated by prenatal screening, with some turning to scepticism about the risk, with others indicating aspiration towards control (Burton-Jeangros et al. 2013).

Some, such as Chen and Wasserman (2017), have argued for an unrestricted approach to prenatal whole-genome sequencing, because this enhances the autonomy and choice of prospective parents. These approaches focus on NIPT through the lens of personal utility (e.g. increasing choice) rather than clinical utility. However, others such as Ravitsky et al. (2017), argue that providing unrestricted options can actually frustrate informed choice rather than enhance it. As has previously been discussed, this is because a whole-genome approach to NIPT can produce an ‘information overload’, where informed choice is no longer possible (Stapleton 2017).

Ravitsky et al. (2017) argue that Chen and Wasserman’s approach requires parents to be highly educated and highly engaged in the prenatal testing process, assuming a high level of health literacy, which may be unrealistic in practice. They argue that many healthcare professionals do not have the appropriate knowledge of or training in genomics to interpret and return these results.

Furthermore, a key concern is that creating the availability of a prenatal test creates, whether perceived or real, a moral obligation to undergo that test (García, Timmermans and van Leeuwen 2012). The extent of this moral obligation is much-debated; some, such as Clarkeburn (2000), argue that there is a moral obligation to undergo a prenatal test when the parents are at risk of having a child whose life is worse than non-existence. Rhodes (2017) argues that there is an obligation to find out information, when it is possible to do so, that would likely make a significant difference to decision-making. In the early era of prenatal screening, provision of the test has been done with a directive approach, presented as something that parents should do to avoid suffering (Stapleton 2017). However, research in the Netherlands suggests that women do not believe they have a moral obligation to do the test, and that it is a personal option that goes beyond parental responsibilities (García, Timmermans and van Leeuwen 2012).

However, considering that there may already be a lack of support for the provision of NIPT for non-medical traits (Bowman-Smart et al. 2019a), it is less likely that women will perceive there to be a moral obligation for them to undertake testing. Chen and Wasserman (2017) argue that “in a pluralistic society in which reproductive choices are regarded as quintessentially private”, where values and circumstances vary widely, it is unlikely that there would be a standard set as to what a ‘reasonable prospective parent’ would do. Furthermore, this objection contains elements of paternalism. Wyatt (2001) argues that instead of paternalism, the mother should be recognised as an ‘expert’ with specialist knowledge in her own unique concerns, alongside the medical professional as ‘expert’ – thus producing an expert-expert relationship.

#### Negative impacts

Some objections rest on the negative impacts that such testing could have on a variety of parties involved in the prenatal testing process. These negative impacts might include psychosocial impacts on the prospective parents (such as increase in anxiety or stress) and negative impacts on society (such as by propagating discriminatory attitudes). If the pregnancy is continued, there may be other negative impacts on the future child (such as the violation of a ‘right to an open future’, or the pressure of expectations) and negative impacts on relationships and the family (such as commodifying the parent-child relationship and distorting bonding).

There are a range of possible negative psychosocial impacts on the prospective parents. For example, after a prenatal diagnosis of a sex chromosome aneuploidy, parents reported higher levels of depression and anxiety (Riggan, Close and Allyse 2020). A 2015 systematic review of anxiety and screening for trisomy 21 found that anxiety increased upon receipt of a high-chance prenatal screening result, although it came back to normal levels if a normal diagnostic result was subsequently received (Lou et al. 2015). One of the reasons reported in the literature for seeking out NIPT is a desire for reassurance and peace of mind (Bowman-Smart et al. 2019b). However, increasing the scope of NIPT to include a wider range of traits means that parents might receive a large amount of information, not all of which is ‘reassuring’. As previously stated, it is likely that any fetus screened in this way has an increased chance of some trait or condition. Thus, the ‘reassuring’ aspect of receiving a low-chance result may cease to be the case. Furthermore, this focus on ‘reassurance’ through NIPT has been criticised, because it may mean prospective parents do not prepare themselves for a high-chance result (Mozersky 2015).

How information is communicated can impact anxiety around prenatal screening. For example, a study in New Zealand found that receiving information about prenatal testing via a video, as opposed to written or audio communication, was associated with greater distress in individuals prone to anxiety (Muller and Cameron 2014). The negative psychosocial impacts on the parents must be weighed against their stated desires to pursue screening and the importance of making fully informed decisions. If these impacts are significant and likely, then this may be a reason to not provide NIPT for non-medical traits in routine practice; however, given that there are means to ameliorate distress through communication and genetic counselling, this may not be a good justification to deny parents the access to this information if they specifically decide to pursue it. They may want to know even if they know it may cause increased anxiety, and information important to decision making may provoke anxiety.

However, overall, the empirical literature to date suggests that NIPT users generally report positive experiences, as well as low decisional regret and elements of psychological distress such as anxiety (incidences of which are associated with insufficient knowledge about NIPT or low health literacy) (Lo et al. 2019, Labonté et al. 2019, and van Schendel et al. 2017). One small randomized clinical trial found that NIPT was associated with increased satisfaction and decreased anxiety compared to first-trimester combined screening (Migliorini et al. 2020). A qualitative interview study from the Netherlands found that most participants did not report feeling a ‘pressure to test’ using NIPT (Bakkeren et al. 2020).

Despite this, while the literature reports generally positive experiences, it is vital to listen to those who have had negative experiences with NIPT provision, such as those who do feel the pressure to test, have decisional conflict, and have had negative experiences post-test (Takeda et al. 2018, Hartwig et al. 2019, Bowman-Smart et al. 2019b, Bakkeren et al. 2020). Widening the scope of the test to non-medical traits may change how women experience NIPT, particularly as approaches like polygenic scores provide only uncertain information. Thus, in-depth consideration of how this information would be provided and its psychosocial impact is critical.

There is also the question of negative impacts on the future child. Deans et al. (2015) argue that using NIPT to test ‘for information only’ should not be allowed, because of possible harms to the future child. These harms might come in a variety of forms. For example, there is the concept of a ‘right to an open future’, where a child (or future child) has the right to not have their genetic information known until such point as they can make their own autonomous decisions regarding this information (Bredenoord, de Vries and van Delden 2014). However, the ‘right to an open future’, as it was originally conceptualised by Joel Feinberg, referred to concepts such as *capacities* (Millum 2014). As Rhodes (2017) points out, self-knowledge does not impede things such as capacities, and so the mere possession of genetic information about the child by the parents does not necessarily jeopardise the child’s right to an open future. Furthermore, the very concept of a right to an open future has also been critiqued (Wilkinson 2005).

Another harm that may befall the child relates to the expectations the parents gain from genetic information about non-medical traits. For example, if the fetus is determined to have a polygenic score that indicates an increased chance for high cognitive ability, the parents may place increased pressure on the child to ‘live up to expectations’ – the ‘living in the shadow’ objection. Similarly, if the fetus has an increased chance of lower cognitive ability, than the parents may not expect the child to achieve very highly, and then not invest as much in their child’s development. However, it is very possible that parents who would hold these expectations, would hold them in any case, based on assessments of their child from a young age.

There may also be negative impacts on the family and parent-child relationships. Sandel (2007) argues that allowing selection for ‘enhancement’ could make parental love conditional, distorting the parent-child relationship and reducing parents’ openness to the unbidden. There is the related concern that prenatal diagnosis ‘commodifies’ the fetus and converts the child into a sort of product that the parents can choose or reject (Rothman 1985).

There may also be negative impacts on society; allowing NIPT for non-medical traits might ‘express’ a negative attitude about people who have/do not have the relevant traits; this is an extension of the disability critique known as the expressivist objection (Hofmann 2017). Ravitsky et al. (2017) also argue that broadening the scope of NIPT and allowing parents to set their own threshold for what they consider relevant, might result in ‘broadening eugenic attitudes’. Indeed, NIPT in its current form – i.e. as a screening tool for trisomy 21 – has been described as a form of ‘contemporary eugenics’ (Thomas & Rothman 2016). There are several responses to this approach; first, we may question whether individual decisions made in the private sphere propagate negative attitudes on the societal level (i.e., questioning whether these harms exist). Second, we may recognise that even if harms do exist, they do not necessarily override the parent’s right to make autonomous decisions about prenatal testing (Edwards 2004). Thirdly, we should anyway attempt to change such attitudes in society.

#### Implementation

Related to these previous objections are general concerns around implementation. One common concern with NIPT is that it may be ‘routinised’ – that is, when it is presented as ‘just a blood test’ women may feel that it is a standard, necessary test that they should undergo as part of routine care (Lewis, Silcock and Chitty 2013, Griffin et al. 2017, Cernat et al. 2019). This relates to the previous discussion on informed choice and pressure to test.

Thus, the concern is that introducing NIPT for non-medical traits would similarly lead to women taking the test as a matter of course without reflecting on the implications that the results could have. However, a response to this is that there are many ways to implement access to NIPT for non-medical traits, and test implementation can be done in a way that does not ‘routinise’ the test. For example, rather than being part of a ‘standard’ aneuploidy test done on a population level that might be publicly funded, as in countries such as Belgium (Kostenko et al. 2018), NIPT for non-medical traits would only be accessed if specifically requested, and/or possibly user pays.

However, making certain information from NIPT only available if specifically requested may create inequities of access between women who are well educated on prenatal testing options and women who are not. Nonetheless, if it were implemented in a way that required increased resources per patient, it may not be economically viable. A recognised issue with expanding the scope of NIPT is that it might result in a greatly increased demand for services such as genetic counselling, which may be necessary to facilitate informed choice and prevent routinisation (Chen and Wasserman 2017). On the other hand, the introduction of NIPT has resulted in resource savings through a significant decrease in rates of invasive testing (Robson & Hui 2015, Kostenko et al. 2018, van den Bogaert et al. 2021). Overall, an objection from routinisation in this context does depend highly on the implementation model that is proposed.

## The case for

Some of the positions in favour of using NIPT to screen for non-medical traits have been briefly raised in response to arguments in the previous section. Here we critically discuss in further depth some of the key arguments put forward to defend the case for NIPT for non-medical traits.

### Reproductive liberty and autonomy

One argument in favour of allowing NIPT for non-medical traits stems from the concepts of reproductive liberty and autonomy. From this perspective, not allowing prospective parents to access NIPT for non-medical traits would violate their reproductive liberty and prevent them from making autonomous (self-directed) choices with respect to their reproduction.

Harris (2005) puts forward a libertarian argument for reproductive liberty that argues that as long as the harms are not of ‘sufficient seriousness’, then parents should have the liberty to access reproductive technologies. Therefore, unless the harms (perhaps to the future child, disabled people, or society) are based on strong evidence and are grave enough, there is no reason to restrict access to NIPT for non-medical traits. Boyle and Savulescu (2003) argue that the onus is on those who wish to restrict access to prenatal testing to demonstrate that it would have harms.

However, Hall (2013) argues that misinformation around prenatal screening results and mistaken beliefs in genetic determinism threatens reproductive liberty. Thomas et al. (2021) have also argued that consent and choice is ‘not sufficient to justify a procedure of questionable clinical utility’.

As mentioned earlier, Chen and Wasserman (2017) argue for an unrestricted approach to prenatal whole-genome sequencing as a means of enhancing reproductive autonomy. They position themselves against those who suggest that access to NIPT should be restricted to conditions based on medical severity (i.e. framing prenatal screening through the lens of clinical utility). They argue that parents should be able to access whatever information that they regard as relevant to their decisions about their pregnancy, although they do place a restriction based on *probability* (i.e., information with low predictive value should not be disclosed). Their framework also requires a process to ensure informed consent, which involves a mandatory presentation and prenatal care visit. As previously outlined, Ravitsky et al. (2017) object to this framework on the basis that too much information would be produced for the framework to work. Allyse et al. (2017) also criticise this framework on the basis that it is unrealistic in terms of implementation, and that it is overly optimistic and relies on idealised circumstances.

We might consider these related concepts – reproductive liberty and autonomy - in two contexts. If NIPT for non-medical traits is used simply to ‘find out’ the information, which Deans et al. (2015) argue against, then the encroachment on the parents’ autonomy may be more justified as the benefit to them is minimal (and assuming there are harms to the future child or others) – the personal utility appears to be less than if they were using this information for reproductive decision-making. However, if NIPT for non-medical traits is used for the purposes of selective reproduction (i.e., TOP), then reproductive autonomy is more relevant, because the parents intend to use the information to make a particular decision about reproduction.

### The concept of ‘non-medical’ traits

In this text, we have repeatedly referred to testing for sub-clinical impacts on traits such as cognitive ability (i.e., cognitive ability within the normal range) as ‘non-medical’ uses of NIPT. However, a key question is whether this distinction between medical and non-medical uses of NIPT is conceptually coherent and useful.

There are several different ways we can conceptualise disease, but as an example, one prominent model is something that causes statistically sub-normal functioning (Boorse 1977, Schwartz 2014). This is not the only way to conceptualise disease, but it is a dominant narrative in culture and medicine. In this framework, to determine whether something is a disease, we examine the functioning of the organism and work backwards. A genetic variant is pathological when it is causally associated with statistically sub-normal functioning (i.e., disease). From this view, pathology is inextricable from concepts of normality.

However, there are many things that cause a decrease in functioning – such as a mild impact on cognitive ability – where we do not require the functioning to reach a sub-normal level before we view an intervention as warranted. An example would be the presence of heavy metals in the environment. Prenatal exposure to lead can result in a reduction of approximately 4 IQ points in children by the age of 7 years (Guo et al. 2020). A reduction of 4 IQ points would be unlikely to lead to sub-normal functioning or intellectual disability. However, it is nonetheless harmful, and measures are undertaken to remove heavy metals from the environment in the name of public health. Thus, we can frame screening for a sub-clinical impact on cognitive ability as ‘medical’.

If a pregnant woman has reason to avoid exposure to lead to avoid a mild decrease in her child’s cognitive ability, then this suggests that a mild decrease in her child’s cognitive ability is meaningful to her in some way. Thus, it may be meaningful to her to engage in prenatal testing for a mild decrease in her child’s cognitive ability and selectively terminate. Both scenarios result in the prevention of a child with a mild decrease in cognitive ability. Of course, in the latter scenario (the termination), she ends up with no child at all. Thus, it is clear that for the latter scenario to occur, the impact on her child would likely have to be of greater importance to her than it would if she simply had to avoid lead.

### Procreative beneficence

There is a view that the parents not only should be able to access NIPT for non-medical traits, but may in fact also be morally obligated to selectively terminate based on the information they receive. This view is the principle of ‘procreative beneficence’ outlined by Savulescu (2001). This principle states that we have good reasons to select the child who is most likely to have the highest level of welfare or well-being. It is most intuitive in the context of pre-implantation genetic testing, where a choice of embryo *must* be made. In the context of selective TOP, where NIPT would be applied, this principle would primarily apply if the parents have the option of having another child (e.g., by getting pregnant again) and TOP did not represent a significant cost to them.

If we consider the example of decreased cognitive ability, we can see how this principle might apply. Decreased cognitive ability may have an impact on welfare. Decreased cognitive ability is associated with a range of negative outcomes. There is evidence to suggest that lower cognitive ability is associated with an increased risk for suicidal behaviour, although the extent of this effect is debated (Hansson Bittár, Falkstedt and Sörberg Wallin 2020, Sörberg et al. 2013, Allen, Bozzay and Edenbaum 2019, Cha et al. 2019), and it is predictive of increased all-cause mortality (Calvin et al. 2010, Christensen et al. 2016, Calvin et al. 2017). Lower cognitive ability is also associated with an increased risk for depression (Koenen et al. 2009, Hung et al. 2016), psychological distress (Gale et al. 2009), violent behaviour (Jacob, Haro et al. 2019), incarceration (Freeman 2012), disability (Jacob, Smith et al. 2019) and decreased health in general (Wrulich et al. 2013). Conversely, higher cognitive ability may be associated with an increased risk for certain psychiatric disorders, such as bipolar disorder (MacCabe et al. 2010, Sørensen et al. 2012, Gale et al. 2013). Decreased cognitive ability is also associated with decreased happiness and mental well-being (Ali et al. 2013, Cheng and Furnham 2014, Ahmed, Kesavayuth and Zikos 2018). Of course, as outlined in the previous discussion of polygenic scores, an association does not necessarily indicate a causal relationship. Thus, there may be several explanations behind these associations that do not relate to biological or genetic factors. Many of these associations may be caused by social factors.

Furthermore, as Savulescu (2007) argues, traits such as cognitive ability can be considered ‘general purpose means’ or ‘all-purpose goods’ – that is, they are traits that are valuable regardless of how good a person’s life is. Therefore, from this perspective, the prospective parents have a *pro tanto* obligation to use NIPT for, and select against, traits such as a sub-clinical impact on decreased cognitive ability. However, because it is *pro tanto*, this means that such a principle would need to be balanced against other things, such as the possible impact of TOP; this principle was originally envisioned in the context of embryo selection, where such countervailing factors may be less important.

There are multiple critiques of the position that we are morally obligated to select the best child. There is extensive discussion of procreative beneficence in the literature, and this is a very brief summary of some key critiques. One critique is that it is impossible to determine what is the ‘best’ life and may depend on competing priorities (e.g. happiness vs physical well-being and so on, or in the case of pleiotropy that we have explored earlier, creativity vs decreased risk of schizophrenia) (Bennett 2014). Sparrow (2007, 50) describes attempts to determine what is the ‘best life’ for the purposes of applying procreative beneficence as “at best extremely controversial, if not ultimately impossible.” However, Savulescu argues that even if it is not possible to determine definitively what is overall a ‘best life’, we still have good reasons to think certain traits are more likely to make a life go well (Savulescu 2007).

Other critiques include that there is no person-affecting harm in a selective reproduction scenario, and thus there is no moral obligation to select (Bennett 2014).; however, there may be impersonal harms that produce the moral obligation (Savulescu 2001). Sparrow (2007) also argues that the logical extension of procreative beneficence is that there is one type of the ‘best’ child that parents have the obligation to bring into the world, and the features of that child will be determined partially by societal discrimination or oppression (e.g. a girl’s life, on balance, is less likely to go well than a boy’s due to sexism). This, along with the force of the obligation generated by procreative beneficence, may result in something very similar to the ‘old eugenics’ of the 1930s (Sparrow 2007, 54).

## Conclusion

Decision-making around prenatal screening should not rest solely on what is possible. Soon, many more things will become possible – and convenient. We thus need to think critically about what kind of information should be available to prospective parents. A focus on pathology is unproductive because we are not talking about patients to be treated. We are talking about parents deciding what lives they want their children to have. Their decision-making encompasses a scope far beyond medical considerations.

This paper is a summary and critical discussion of the case for and against the use of NIPT for non-medical traits. What we emphasise here is that there is currently a lack of consistency in decision-making around prenatal screening, and how we view and assess different traits. There will be new challenges from techniques, such as those using polygenic scores. We must develop a coherent and consistent approach that focuses on the morally relevant features of a trait, and the context in which the prospective parents make their choices. To do so, we must reflect not only on the ethical arguments outlined here, which can form the basis of informing this process, but also involve the views of the people who will be using these services – that is, prospective parents. Similar technologies are already being marketed (e.g., for embryo screening). We must develop a consistent ethical framework *before* these technologies become widespread.
